# Reconstruction error based deep neural networks for coronary heart disease risk prediction

**DOI:** 10.1371/journal.pone.0225991

**Published:** 2019-12-05

**Authors:** Tsatsral Amarbayasgalan, Kwang Ho Park, Jong Yun Lee, Keun Ho Ryu

**Affiliations:** 1 Database and Bioinformatics Laboratory, School of Electrical and Computer Engineering, Chungbuk National University, Cheongju, Korea; 2 Faculty of Information Technology, Ton Duc Thang University, Ho Chi Minh City, Vietnam; 3 College of Electrical and Computer Engineering, Chungbuk National University, Cheongju, Korea; College of Bioinformatics Science and Technology, CHINA

## Abstract

Coronary heart disease (CHD) is one of the leading causes of death worldwide; if suffering from CHD and being in its end-stage, the most advanced treatments are required, such as heart surgery and heart transplant. Moreover, it is not easy to diagnose CHD at the earlier stage; hospitals diagnose it based on various types of medical tests. Thus, by predicting high-risk people who are to suffer from CHD, it is significant to reduce the risks of developing CHD. In recent years, some research works have been done using data mining to predict the risk of developing diseases based on medical tests. In this study, we have proposed a reconstruction error (RE) based deep neural networks (DNNs); this approach uses a deep autoencoder (AE) model for estimating RE. Initially, a training dataset is divided into two groups by their RE divergence on the deep AE model that learned from the whole training dataset. Next, two DNN classifiers are trained on each group of datasets separately by combining a RE based new feature with other risk factors to predict the risk of developing CHD. For creating the new feature, we use deep AE model that trained on the only high-risk dataset. We have performed an experiment to prove how the components of our proposed method work together more efficiently. As a result of our experiment, the performance measurements include accuracy, precision, recall, F-measure, and AUC score reached 86.3371%, 91.3716%, 82.9024%, 86.9148%, and 86.6568%, respectively. These results show that the proposed AE-DNNs outperformed regular machine learning-based classifiers for CHD risk prediction.

## Introduction

According to the statement by the World Health Organization (WHO), CHD is said to be one of the fatal disorders in the world, and as for 2016, an estimated 15.2 million people died from CHD and stroke [1]. South Korea is equally considered to be one of the countries in which heart disease is higher, being ranked second of all deaths [2]. Risk factors of developing CHD include bad habits, such as lack of physical activity, poor diet, smoking and stress [3, 4]. If it reaches the end-stage, the most advanced treatments are required to be conducted, including stent surgery that helps keep coronary arteries open and reduce the chance of a heart attack, and coronary artery bypass grafting that improves blood flow to the heart, and heart transplant [5, 6]. In the early stage, it is possible to prevent suffering from CHD by giving advice and appropriate medicines. However, it is difficult to make precise diagnose at the earlier-stage of CHD [7, 8]; hospitals use many kinds of clinical tests, such as electrocardiogram, coronary computed tomography angiogram, echocardiogram and blood tests [9].

In recent times, data mining based classification techniques have been used in the research works, predicting the risk of CHD [10–16]. Data mining is a process of automatically discovering useful information in large data repositories and provides an opportunity to predict the outcome of further observation [17]. If data have labels revealing high risky or normal, classification techniques are used. In [15, 18], the authors have compared several classification models: Decision tree (DT), Naïve Bayes (NB), K-nearest neighbors (KNN), Neural network (NN), and Support vector machine (SVM). According to the findings from these researches, the SVM algorithm showed the highest accuracy, with Kim et al., 2015 reaching higher accuracy (69.51%) than SVM by using the Fuzzy Logic and Decision tree on the sixth Korea National Health and Nutrition Examination Survey (KNHANES) dataset. In [14, 16, 19, 20], DNN based CHD prediction modesl have been proposed and, Atkov et al., 2012 has built 10 different prediction models by different risk factors—each model has two hidden layers with 4 neurons—the accuracy reached 93% on 487 patients’ data in Central Clinical Hospital No. 2 of Russian railways. In [19, 20], the authors have done feature correlation analysis, and hidden layers of DNN were connected based on the correlation results; the accuracy and AUC score of the DNN with feature correlation analysis were 83.9% and 79.0% on the sixth KNHANES dataset.

By this research work, we have proposed the RE based DNNs; we have aimed to improve the performance of DNN by extracting the RE based feature from the deep AE neural network model. In most cases, AE is used as a dimensionality reduction technique to project high dimensional space into lower-dimensional space. Dimension reduction techniques are mostly used to avoid the curse of dimensionality problem before cluster or classification algorithms [21–24]. The novelty of our research work is that we used RE instead of low dimensional space of deep AE model to extract the new feature. Reconstruction is a process of transforming the lower-dimensional representation of source data into its original dimension. RE is estimated by a difference between source data and reconstructed data. We have evaluated the proposed AE-DNNs on a 6-years KNHANES dataset; it presented higher performance than the previous studies with the same dataset as well as other machine learning-based algorithms.

## Methodology

**Fig 1:**
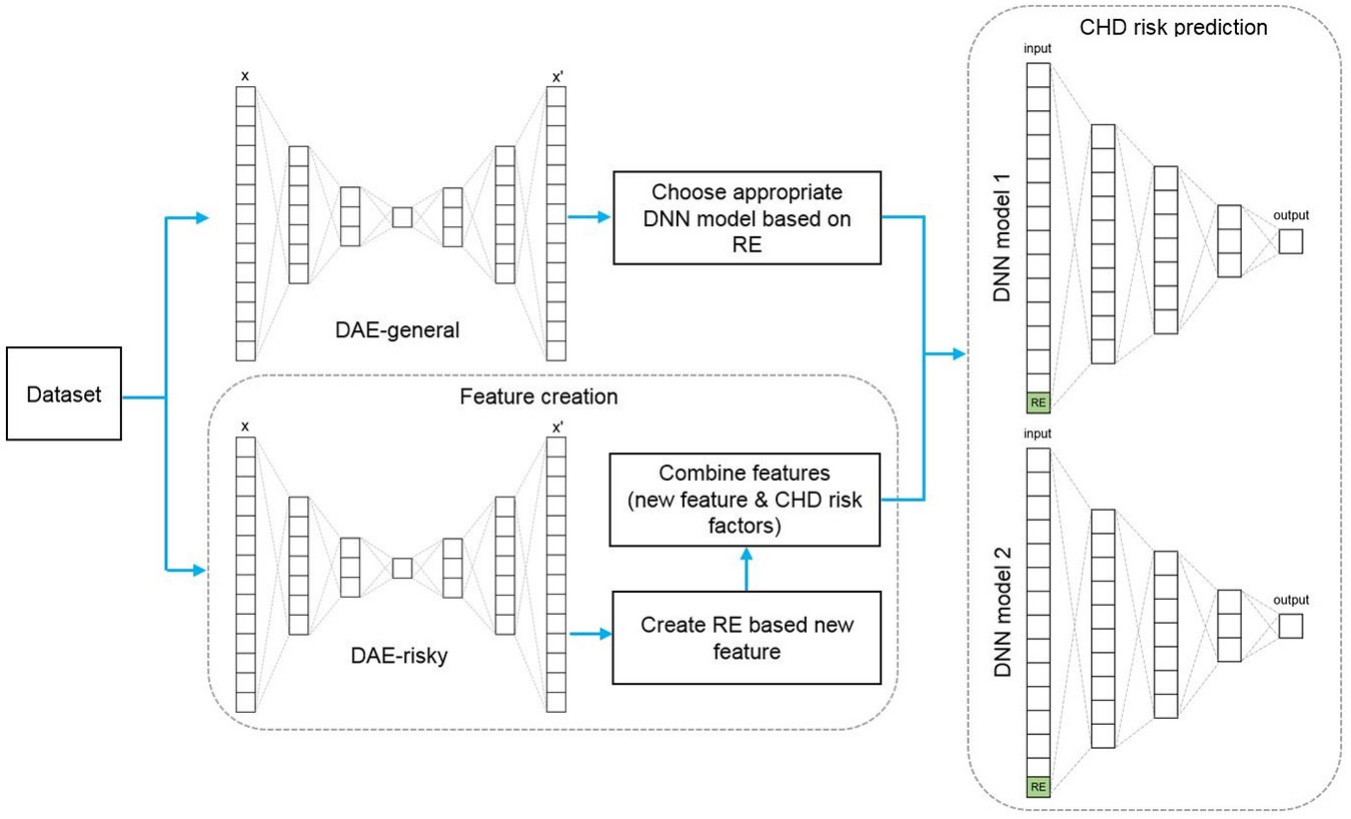
General architecture of the proposed AE-DNNs method. DAE, deep autoencoder; DNN, deep neural network; RE, reconstruction error; CHD, coronary heart disease.

In this section, we will describe details of how to create the new feature using RE and to predict the risk of developing CHD by DNNs. Our proposed approach has two fundamental functions: feature extraction and CHD risk prediction. The general architecture of the proposed method is shown in Fig 1.

### RE based feature extraction

As mentioned above, AE is a technique for reducing the dimension. However, we have created the RE based new feature using this technique and have shown its results in the results and discussion section.

**Fig 2:**
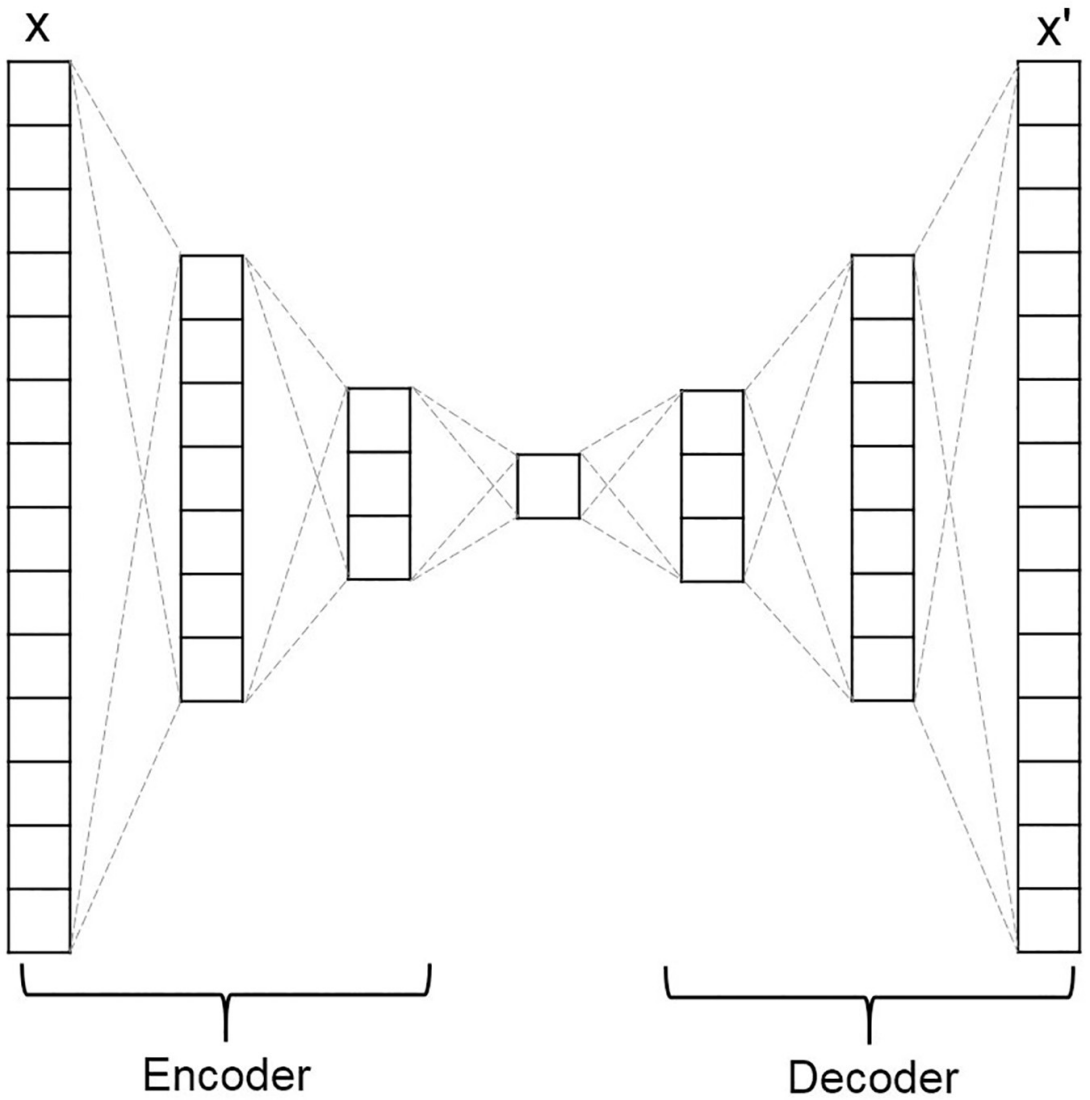
A simple autoencoder neural network architecture based on a fully-connected layer.

AE is one kind of neural network (NN) [25] as well as being an unsupervised algorithm that learns to copy its input (x_1_ … x_n_) to its output (x’_1_ … x’_n_) as close (x_i_ = x’_i_) as possible by reducing the difference between inputs and outputs [26]. Thus, the number of input neurons equal to the number of output neurons in AE. In general, the structure of AE is similar to NN with one hidden layer, at least, whereas AE is distinguished from the NN for predicting output label by the aim of input reconstruction. As shown in Fig 2, AE consists of the encoder and decoder parts; firstly, it projects input x to a lower dimension that works in encoder part, then it reconstructs output x’ from the low dimensional projection that works in decoder part. In other words, the learning process of AE is that it compresses the input into a lower-dimensional space called latent space and uncompresses back the compressed data into the output that closely matches the original data. Then, it calculates a difference between the input and reconstructed output and changes the weights of the network to reduce this difference.

AE takes an input vector *x* and maps it into a hidden representation *h*, then the hidden representation *h*, sometimes called the latent space, is mapped back to a reconstructed vector *x’*. The following equation describes an AE:

$$\begin{array}{c} \begin{array}{c} h=a(wx+b)\\ x^{\prime }=a^{\prime }\left(w^{\prime }h+b^{\prime }\right) \end{array} \end{array}$$ (1)

where *w* and *w’* are the weight matrices, *b* and *b’* are the bias vectors, and *a* and *a’* are the activation functions. The parameters of the AE model are optimized to minimize the average RE, as shown in Eq 2:

$$MSE=\frac{1}{n}\sum_{i=1}^{n}{\left(x_{i}-x_{i}^{\prime }\right)}^{2}$$ (2)

where *MSE* is the mean squared error, *n* is the number of a dataset, *x* is the input, and *x’* is the output.

In this study, we used DAE-general and DAE-risky two deep AE models, showing in Fig 1. The DAE-risky model learns from the only high-risk subset of the training dataset for the RE based feature extraction. The DAE-general model is used to choose an appropriate classifier in CHD risk prediction module that trains on the whole training dataset. In this section, we will describe how the DAE-risky model is employed in the feature extraction process. It is possible to identify well which differentiation of RE is risky or normal by training the AE model on the only risky dataset. In other words, if we give a person’s data who has low CHD risk as an input of DAE-risky model, the RE will tend to be higher because the model did not learn from the low risk dataset. Algorithm 1 shows the steps of how to do feature extraction, one of two fundamental functions of our proposed approach.

**Algorithm 1** Feature extraction.

1: Set of points $X,\left\{X_{i}^{n}=1\right\}$

2: Select *risky* subset from *X* dataset

3: Train *DAE*_*risky* on the *risky* subset

4: *Z* ← []

5: **for** *i* ∈ 0…len($\hat{X})$ **do**

6:  $\hat{X_{i}}\leftarrow DAE\_risky.predict\left(X_{i}\right)$

7:  $Z_{i}\leftarrow {\left(\hat{X_{i}}-X_{i}\right)}^{2}$

 **return** *Z*

First, all risky subset is selected from the *n* number of training dataset and then the DAE-risky model is trained on the selected subset. According to Algorithm 1, the RE based new feature is calculated by a squared difference between initial input and its reconstructed output. The equation of the proposed deep AE neural network can be written in the vectorized form:

$$\begin{array}{c} \begin{array}{c} x^{l}=tanh(w^{l}tanh(w^{l-1}tanh(w^{l-2}relu(w^{l-3}relu\left(w^{l-4}relu\left(w^{l-5}x+b^{l-5}\right)+b^{l-4}\right)\\ +b^{l-3})+b^{l-2})+b^{l-1})+b^{l}) \end{array} \end{array}$$ (3)

where *tanh* and *relu* are the activation functions, *w^l^* and *b^l^* are the weight matrix and the bias vector for each layer, *l*, and *x* is the input vector.

### DNNs for CHD risk prediction

**Fig 3:**
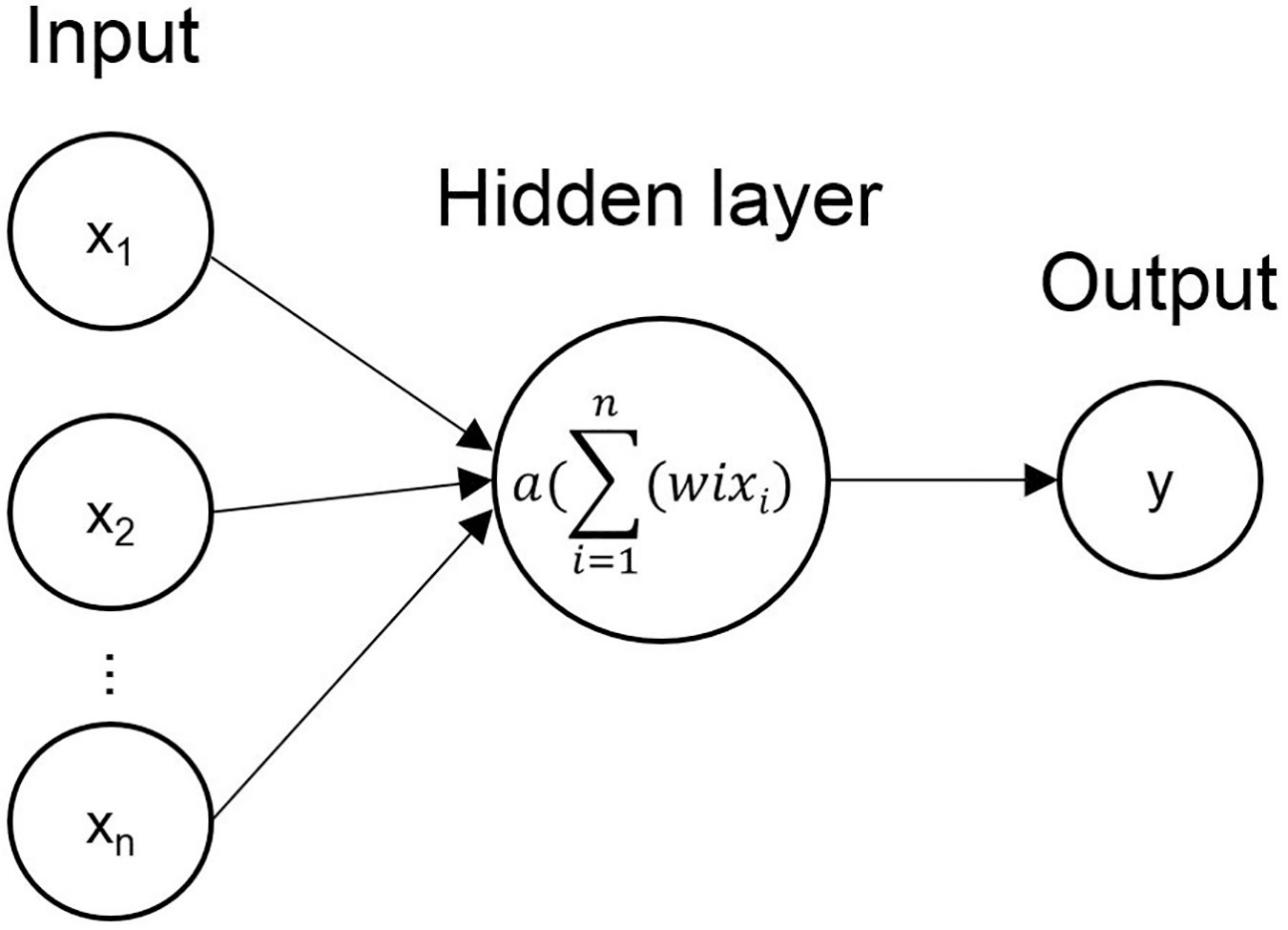
A simple neural network architecture with one hidden layer.

NN is mostly used to predict output labels from the input, consisting of an input layer, hidden layer, and output layer [27]. The input layer is composed of neurons that describe input features whereas neurons in hidden and output layers receive a result of activation function that converts the weighted summation of the neurons of the previous layer, as shown in Fig 3. NN learns by changing the weights of each neuron to reduce an error between target class y and predicted class y’.

In this study, we proposed two NN classifiers trained on different groups of a dataset. In practically, a dataset can include a subset which is higher variance than the most dataset and that highly biased dataset degrades the performance of classification techniques. Therefore, we isolated a highly biased dataset from the common dataset using the DAE-general model that learned from the whole training dataset. The data that is different from most dataset gives higher RE than common data on the DAE-general model. As shown in Fig 1, we used two independent deep AE models. The DAE-general is used for data grouping and and selection of the CHD risk prediction model. The DAE-risky is used for feature extraction. The only difference is that DAE-risky trained on risky subset while the DAE-general trained on the whole training dataset. For estimating the data splitting threshold, first, we calculated reconstruction errors of the training dataset by the squared difference between the input and output. The threshold value is estimated by the mean and standard deviation of the reconstruction errors; it can be described as:

$$threshold=\frac{1}{n}\sum_{i=1}^{n}RE_{i}+\sqrt{\frac{1}{n}\sum_{i=1}^{n}(RE_{i}-\frac{1}{n}\sum_{i=1}^{n}RE_{i}{)}^{2}}$$ (4)

where *RE* is the reconstruction error vector, and *n* is the number of elements in RE vector.

After threshold estimation, we partitioned the training dataset into two parts; each part consists of subsets labeled by high-risk and low-risk. The first part contained a dataset with high RE that exceeds the threshold value and the second part consisted of the rest of the dataset. Subsequently, DNN classifiers are trained on each group separately using CHD risk factors with RE based newly created feature. Each of NN classifiers is the same structure that has four hidden layers with neurons 10, 7, 5, and 3, respectively. The input layer consists of 15 neurons, including the selected CHD risk factors, and the RE based feature to predict target variable y.

$$\begin{array}{c} \begin{array}{c} y=sigmoid(w^{l}relu(w^{l-1}relu(w^{l-2}relu\left(w^{l-3}relu\left(w^{l-4}+b^{l-4}\right)+b^{l-3}\right)\\ +b^{l-2})+b^{l-1})+b^{l}) \end{array} \end{array}$$ (5)

where the output layer uses the *sigmoid* activation function, and all of the hidden layers use the *relu* activation function, *w^l^* and *b^l^* are the weight matrix and the bias vector for each layer, *l*, and *x* is the input vector.

In the CHD risk prediction process, first, test data is given as an input of the trained DAE-general model, and its RE is calculated. If the RE exceeds the threshold, the DNN model 1 that trained on data group with high RE will be used; otherwise, the DNN model 2 that trained on data group with lower RE will be used to predict the class label, as shown Fig 1.

## Experimental Study

**Fig 4:**
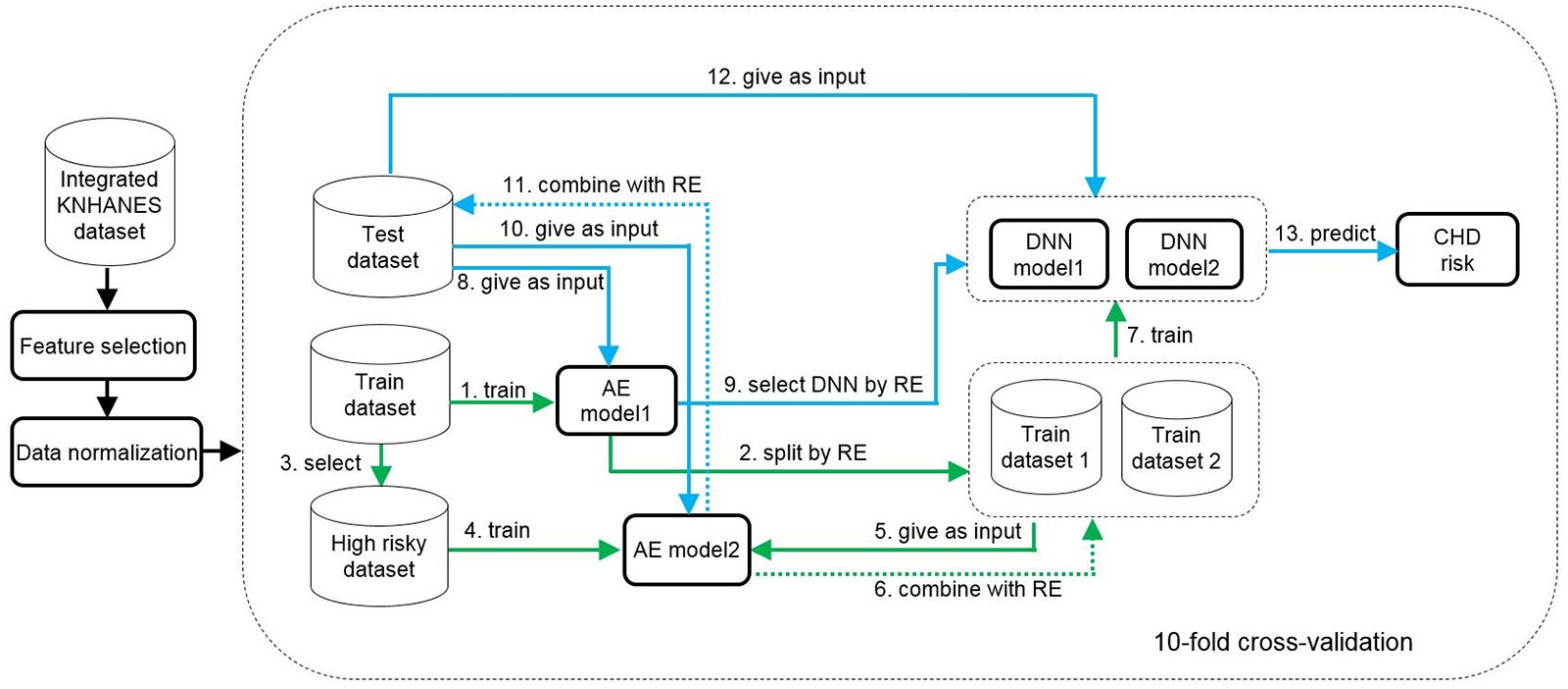
Design of experimental study for the AE-DNNs. Steps 1-7 (green line) represent model training, and steps 8-13 (blue line) show model evaluation. KNHANES, Korea national health and nutrition examination survey; AE, autoencoder; RE, reconstruction error; DNN, deep neural network; CHD, coronary heart disease.

We show the result of the AE-DNNs by comparing with machine learning-based NB, RF, KNN, DT, and SVM algorithms. The design of the experimental study for the proposed method is shown in Fig 4.

### Data pre-processing

KNHANES is the Nationwide Program to evaluate Koreans’ health and nutritional status. It consists of 3 parts: Health examination, health interview and nutrition survey. It has been continuously collected since 1998 [28]. The KNHANES datasets are released for public use within one year of the end of each survey year [29, 30].

**Fig 5:**
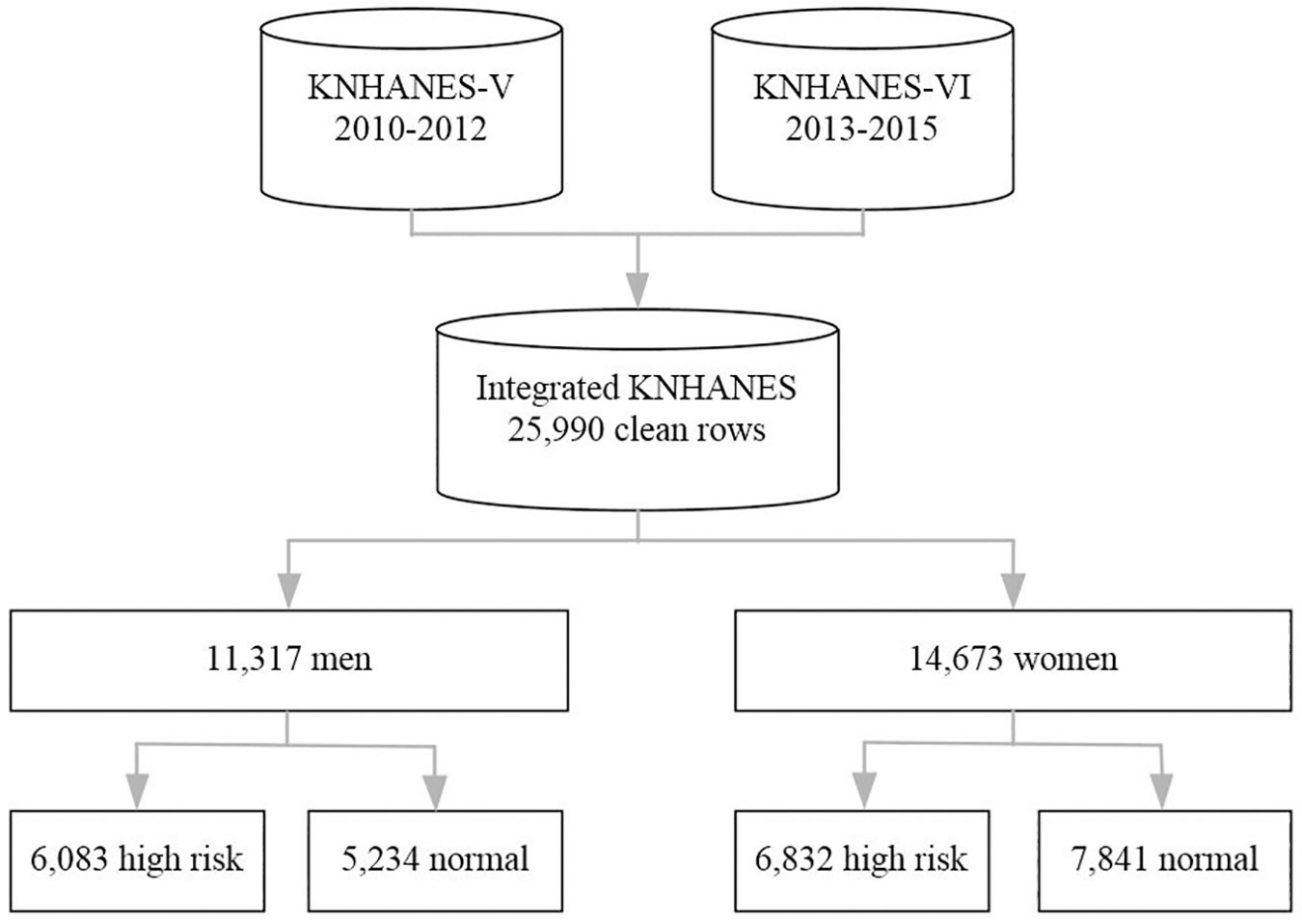
Integrated KNHANES dataset.

We have analyzed samples spanning the years 2010-2015. After removing missing valued rows, a total of 25,990 records include 11,317 men, and 14,673 women were used in our experiment. There are 12,915 records of high-risk people and 13,075 records of low-risk people in the dataset. Fig 5 illustrates the integrated KNHANES dataset.

Selecting valuable features from the experimental dataset plays an important role in building accurate prediction model. It not only reduces the space and computation complexity but also improves the performance of the classification model. We selected 14 features that influenced in CHD from the total number of 423 features of a health interview, health examination, and nutrition survey using an extremely randomized tree classifier in the scikit-learn machine learning package in Python on all variables and chi-square test on categorical variables. The general descriptions of the selected features are shown in Table 1.

**Table 1:** Description of the selected features.

Variable name	Low risk (13,075 records)	High risk (12,915 records)
*Age*(*yr*)	41.54 (17.71)	55.96 (15.55)
*Body mass index* (*kg*/*m*2)	22.53 (3.27)	24.61 (3.33)
*Total cholesterol* (*mg*/*dL*)	181.16 (28.55)	194.17 (41.91)
*High* − *density lipoproteincholesterol* (*mg*/*dL*)	54.53 (10.33)	46.23 (12.18)
*Systolic blood pressure* (*mmHg*)	113.28 (14.97)	124.19 (17.41)
*Waist circumference* (*cm*)	76.94 (9.55)	84.40 (9.44)
*Neutralfat* (*mg*/*dL*)	93.24 (48.54)	165.91 (125.52)
*Obesity status*		
1. *Underweight*	1176	254
2. *Normal*	9258	7188
3. *Obesity*	2641	5473
*Life time smoking status*		
1. *Less than* 5 *packs* (100 *units*)	336	224
2. *More than* 5 *packs* (100 *units*)	3755	5200
3. *Never smoked*	7478	7235
8: *Non* − *applicable* (*youth*, *child*)	1501	251
9. *No response*	5	5
*Diabetes*		
1. *Normal*	10678	7427
2. *Fasting blood sugar disorder*	1916	3262
3. *Diabetes*	481	2226
*Knee joint pain status*		
1. *Yes*	835	2070
2. *No*	4035	7204
8. *Non* − *applicable* (*below the 50 years of age*)	8201	3637
9. *No response*	4	4
*Weight change in one year status*		
1. *No change*	7447	8638
2. *Weight loss*	1634	1743
3. *Weight gain*	2481	2269
8. *Non* − *applicable* (*youth*, *child*)	1501	251
9. *No response*	12	14
*Frequency of eating out year status*		
1. *More than twice a day*	1147	735
2. *Once a day*	2182	1606
3. 5 *to* 6 *times a week*	2570	1502
4. 3 *to* 4 *times a week*	1303	1070
5. 1 *to* 2 *times a week*	2804	2883
6. 1 *to* 3 *times a month*	2242	3336
7. *Less than once a month*	825	1781
9. *No response*	2	2
*Marital status*		
1. *Married*, *living together*	8338	9736
2. *Married*, *living separately*	58	85
3: *Bereavement*	566	1576
4. *Divorced*	300	436
8. *Response refused*	374	165
9. *No response*	2	9
88. *Non* − *applicable*	3426	908

The features such as age, knee joint pain status, lifetime smoking status, waist circumference, neutral fat, body mass index, weight change in one year status, systolic blood pressure, total cholesterol, obesity status, frequency of eating out, high-density lipoprotein cholesterol, marital status, and diabetes were used as risk factors of CHD prediction model. Hypertension, dyslipidemia, stroke, myocardial infarction, angina, and hyperlipidemia were used to identify class labels (high-risk or low-risk). In other words, in case one of these 6 disorders is identified, the individual will be considered to have high CHD risk.

In this paper, we presented the DNN based CHD prediction model. NN is a black-box model, it does not give importance of each feature, and any insights on the structure explicitly. Therefore, we trained several DNN models by removing each features one by one from all and then ranked all features by how they affect the DNN model. First, we trained a baseline model with DNN based on all 14 features. Then, features were ranked depends on the difference of accuracy between the baseline model and the newly trained model that eliminated a feature, shown in Table 2. From the table, all features affect the effectiveness of prediction results because all accuracy was decreased when get rid of a particular feature.

**Table 2:** Result of feature ranking.

DNN models	Accuracy	Difference from baseline	Rank
*DNN*(*all features*)	0.8547		
*DNN*(*all features*–*high density lipoprotein cholesterol*)	0.7928	0.0619	1
*DNN*(*all features*–*total cholesterol*)	0.8132	0.0415	2
*DNN*(*all features*–*neutral fat*)	0.8308	0.0239	3
*DNN*(*all features*–*obesity status*)	0.8428	0.0119	4
*DNN*(*all features*–*frequency of eating out*)	0.8477	0.0070	5
*DNN*(*all features*–*diabetes*)	0.8485	0.0062	6
*DNN*(*all features*–*age*)	0.8487	0.0060	7
*DNN*(*all features*–*weight change in one year status*)	0.8511	0.0036	8
*DNN*(*all features*–*systolic blood pressure*)	0.8513	0.0034	9
*DNN*(*all features*–*waist circumference*)	0.8524	0.0023	10
*DNN*(*all features*–*body mass index*)	0.8528	0.0019	11
*DNN*(*all features*–*life time smoking status*)	0.8530	0.0017	12
*DNN*(*all features*–*marital status*)	0.8539	0.0008	13
*DNN*(*all features*–*knee joint pain status*)	0.8541	0.0006	14

Additionally, we retrained DNN models again by removing the lowest-ranked features step by step manner until remain one feature, and results are written in Table 3. As a result, the accuracy of all models is less than the baseline model. Therefore, all selected 14 features were used together for further experiments.

**Table 3:** Results of DNN models with recursive feature elimination.

DNN models	Accuracy	Difference from baseline
*DNN*(*without knee joint pain status*)	0.8541	0.0006
*DNN*(*without previously eliminated features* & *marital status*)	0.8529	0.0018
*DNN*(*without previously eliminated features* & *life time smoking status*)	0.8542	0.0005
*DNN*(*without previously eliminated features* & *body mass index*)	0.8504	0.0043
*DNN*(*without previously eliminated features* & *waist circumference*)	0.8517	0.0030
*DNN*(*without previously eliminated features* & *systolic blood pressure*)	0.8451	0.0096
*DNN*(*without previously eliminated features* & *weight change in one year status*)	0.8457	0.0090
*DNN*(*without previously eliminated features* & *age*)	0.8284	0.0263
*DNN*(*without previously eliminated features* & *diabetes*)	0.8181	0.0366
*DNN*(*without previously eliminated features* & *frequency of eating out*)	0.8112	0.0435
*DNN*(*without previously eliminated features* & *obesity status*)	0.8055	0.0492
*DNN*(*without previously eliminated features* & *neutral fat*)	0.7662	0.0885
*DNN*(*without previously eliminated features* & *total cholesterol*)	0.6936	0.1611

**Fig 6:**
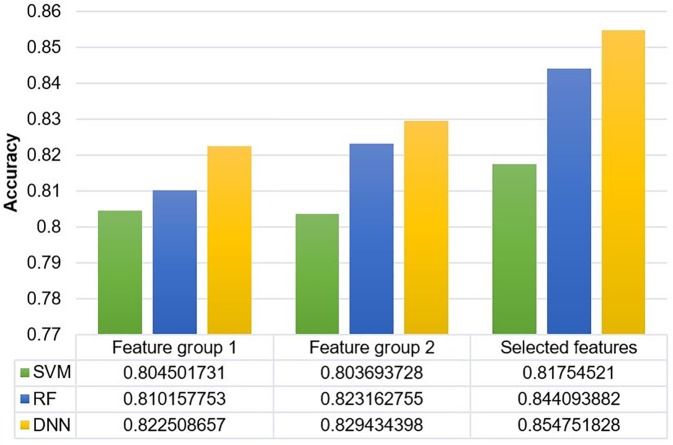
Comparison of the selected features and other guidelines. The feature group 1 is the Framingham risk factors, and the feature group 2 was proposed by [18-20] papers on KNHANES dataset.

The Framingham risk score (FRS) is the multivariable statistical model used to identify the risks of developing CHD based on age, sex, smoking status, hypertension, cholesterol, high-density lipoprotein cholesterol, and diabetes [31]. In [18–20] papers, the authors have built the data mining based models on KNHANES dataset to predict the risk of CHD using Framingham risk factors and several hospital tests. We compared our selected features with the risk factors of these guidelines by RF, SVM, and DNN algorithms, showing in Fig 6.

### Compared methods

We have compared the proposed AE-DNNs to the following supervised machine learning techniques. For the compared algorithms, we have chosen optimal values of input parameters by changing their values until decreasing the performance.

#### NB

The Naïve Bayes is probability-based classification algorithm. It computes the posterior probability for each class label and picks the class label that has the highest probability. In NB, it does not calculate the probability based on combined features, instead, considering all features separately; it is called conditional independence [17].

#### KNN

The k-nearest neighbor algorithm is used for the classification task. In the classification phase, a user defines the value of the k parameter, and an unlabeled instance is labeled by the most frequently occurred class label among the k number of nearest training samples. First, it calculates distances between the unlabeled instance and each training data to find the nearest neighbors. Belongs to the k number of nearest neighbors, a majority voted class label will be assigned to the output label. We have configured the value of the k between 2 and 20.

#### DT

The decision tree classifier is a simple algorithm that has been widely used so far [17]. The goal is to create a model that predicts the value of a target variable by learning simple decision rules inferred from the data. Classification and regression trees (CART) was introduced by Breiman in 1984 and very similar to C4.5, but the main difference is that it supports numerical target value (class label). It builds both classification and regression trees [32]. The classification tree construction by CART is based on the binary splitting of the attributes. We have used “gini” for the gini impurity and “entropy” for the information gain to measure the quality of a split.

#### RF

The random forest is the one kind of ensemble algorithm [17]. It creates several decision tree classifiers on various sub-samples of the dataset and uses averaging to improve the predictive accuracy and control over-fitting [33]. In this research work, we have adjusted the number of trees in the forest between 10 and 150.

#### SVM

The support vector machine is a supervised learning method, and it has shown promising empirical results in many practical applications [17]. It finds a separator line called the hyper-plane that differentiate the classes very well and learns by minimizing the classification error and maximizing the margin. SVM can be extended to non-linearly separable data using kernel function. We have built the SVM model by using kernel functions such as linear, poly, rbf, and sigmoid.

#### PCA-DNNs

The proposed AE-DNNs uses the RE for two kinds of purposes. The first one is RE based feature extraction by the AE model that trained on the high-risk subset of the training dataset. The second one is used to arrange training dataset into two groups based on their RE divergence by the AE model that trained on the whole training dataset. Subsequently, two DNN models learn from these groups. In the prediction process, the second AE model is also employed to choose a proper classification model. Therefore, we have used Principal component analysis (PCA) in place of AE for calculating RE. PCA is a dimension reduction technique that the direction with the largest projected variance is called the first principal component. The orthogonal direction that captures the second largest projected variance is called the second principal component, and so on [34]. We can estimate RE by projecting back a low dimension to original space using PCA.

### Results and discussion

The experiment was done on a computer with i5-8500 CPU, NVIDIA GeForce GTX 1070 Ti graphics card, and 32GB RAM. We have compared our proposed approach to other machine learning-based algorithms by experimenting with total 6-years data that integrated KNHANES-V and KNHANES-VI datasets. All compared algorithms were implemented in Python with Keras, which is a high-level neural networks API, written in Python and capable of running on top of TensorFlow. For AE-DNNs, two DNN classifiers were configured as same as each other. Also, the learning rate to minimize the mean squared error was 0.001, and the Adamax optimizer was used [35]. The batch size was 32, and the number of epochs was 5000. Before train prediction models, we have normalized our dataset. Standardization is a widely used normalization method in machine learning algorithms, and it calculates mean and standard deviation for each attribute of a dataset. Then each value is subtracted by the mean, and the subtracted result is divided by the standard deviation.

The accuracy, precision, recall, specificity, f-measure, and area under the curve (AUC) that represents a summary measure of accuracy is used to evaluate the classifiers’ performance. We have used the methodology of 10-fold cross-validation to measure.

Table 4 shows the results of CHD prediction models that learned from risk factors in Table 1, and the highest values of evaluation scores are marked in bold. The configuration of input parameters is shown in the column of the Algorithm.

**Table 4:** Results of compared algorithms on integrated KNHANES dataset.

Algorithm	Accuracy	Precision	Recall	Specificity	F-measure
*NB*	0.7421	0.7940	0.6493	0.8246	0.7123
*KNN*(*n*_*neighbors* = 19)	0.7919	0.7645	0.8415	0.7364	0.8011
*DT*(*criterion* = ‘*gini*’)	0.7819	0.7833	0.7763	0.7851	0.7797
*RF*(*n*_*estimators* = 110, *criterion* = ‘*gini*’)	0.8442	0.8150	**0.8881**	0.7979	0.8499
*SVM* (*kernel* = ‘*rbf*’)	0.8175	0.8053	0.8358	0.7948	0.8202
*PCA* − *DNNs*	0.8537	0.9012	0.8217	0.8908	0.8595
*Proposed AE* − *DNNs*	**0.8633**	**0.9137**	0.8290	**0.9041**	**0.8691**

As a result, the AE based proposed approach outperformed the PCA based version. For machine learning-based compared algorithms, the RF algorithm showed higher performance than others. In Table 4, the precision of the proposed AE-DNNs increased by 9.86% from the RF algorithm, but the recall decreased by 5.91%. The recall is a fraction of the true positive predictions over the total amount of positively labeled dataset, while precision is a fraction of the true positive predictions among all positive predictions. In other words, recall measures what proportion of actual positives was identified correctly, and the precision evaluates the effectiveness of true positive predictions. However, as recall gives a high score, the number of false true prediction can be increased relatively. That means improving recall typically reduces precision and vice versa. It is difficult to compare models with low precision and high recall or high precision and low recall. Thus, F-measure is used to measure recall and precision at the same time, where the highest F-measure indicates a good result. The specificity is a fraction of the true negative (high risky) predictions over the total amount of negatively labeled dataset. The AE-DNNs improved the specificity by 10.61% from the RF and 7.94% from the NB algorithm. Moreover, AE-DNNs showed the result of the highest accuracy (86.33%) and F-measure (86.91%).

Table 5 shows the Receiver operating characteristic (ROC) curve analysis results. We tested whether observed AUC differs significantly from AUC of 0.5 by Hanley and McNeil test [36]. For all compared algorithms, AUC (<0.000001) was statistically significant. In the case of AE-DNNs, AUC was 0.8665 (95% CI, 85.25-88.07), and it improved the highest AUC of compared algorithms (AUC of RF) by 2.35%.

**Table 5:** Results of the receiver operating characteristic curve analysis of compared algorithms.

Algorithm	AUC	p-value	95%CI
*NB*	0.7370	1.52848E-44	0.7180-0.7561
*KNN*	0.7889	3.5552E-78	0.7715-0.8064
*DT*	0.7807	2.34585E-65	0.7630-0.7984
*RF*	0.8430	9.901E-113	0.8277-0.8583
*SVM*	0.8153	5.15569E-96	0.7989-0.8318
*PCA* − *DNNs*	0.8562	1.5289E-125	0.8417-0.8709
*Proposed AE* − *DNNs*	0.8665	1.4386E-132	0.8525-0.8807

**Fig 7:**
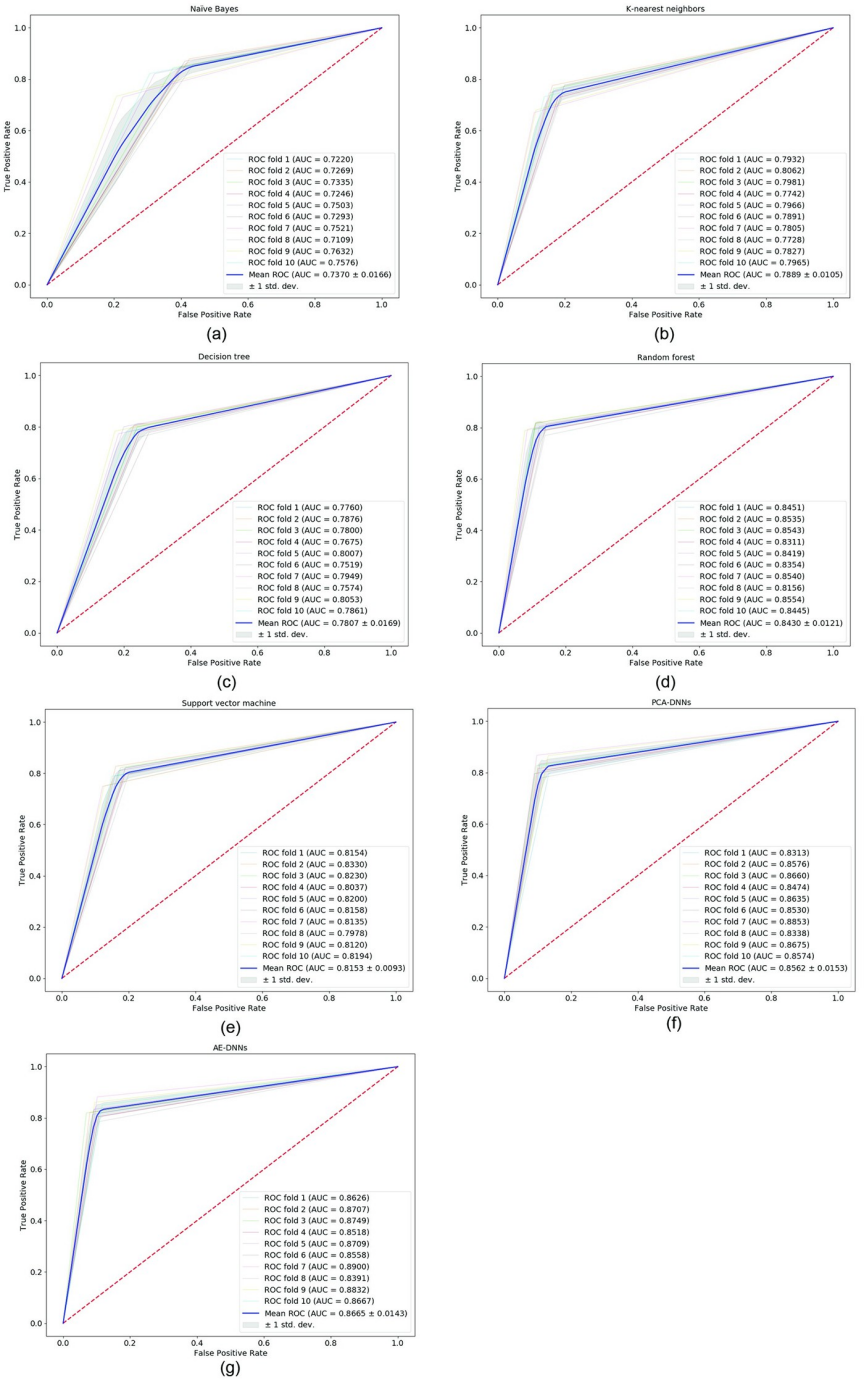
Receiver operating characteristic curves of compared algorithms. (a) Receiver operating characteristic curves of Naïve Bayes algorithm; (b) Receiver operating characteristic curves of k-nearest neighbors algorithm; (c) Receiver operating characteristic curves of decision tree algorithm; (d) Receiver operating characteristic curves of support vector machine algorithm; (e) Receiver operating characteristic curves of random forest algorithm; (f) Receiver operating characteristic curves of principal component analysis based deep neural networks (PCA-DNNs) algorithm; (g) Receiver operating characteristic curves of autoencoder based deep neural networks (AE-DNNs) algorithm.

Fig 7 shows the ROC curves of each compared algorithms on the integrated KNHANES dataset with 10-fold cross-validation distinctly. For 10-fold cross-validation, it is possible to calculate the mean area under the curve, and see the variance of the curve when the training set is split into different subsets. Therefore, we show the ROC curve of each round of cross-validation with variance in Fig 7.

**Fig 8:**
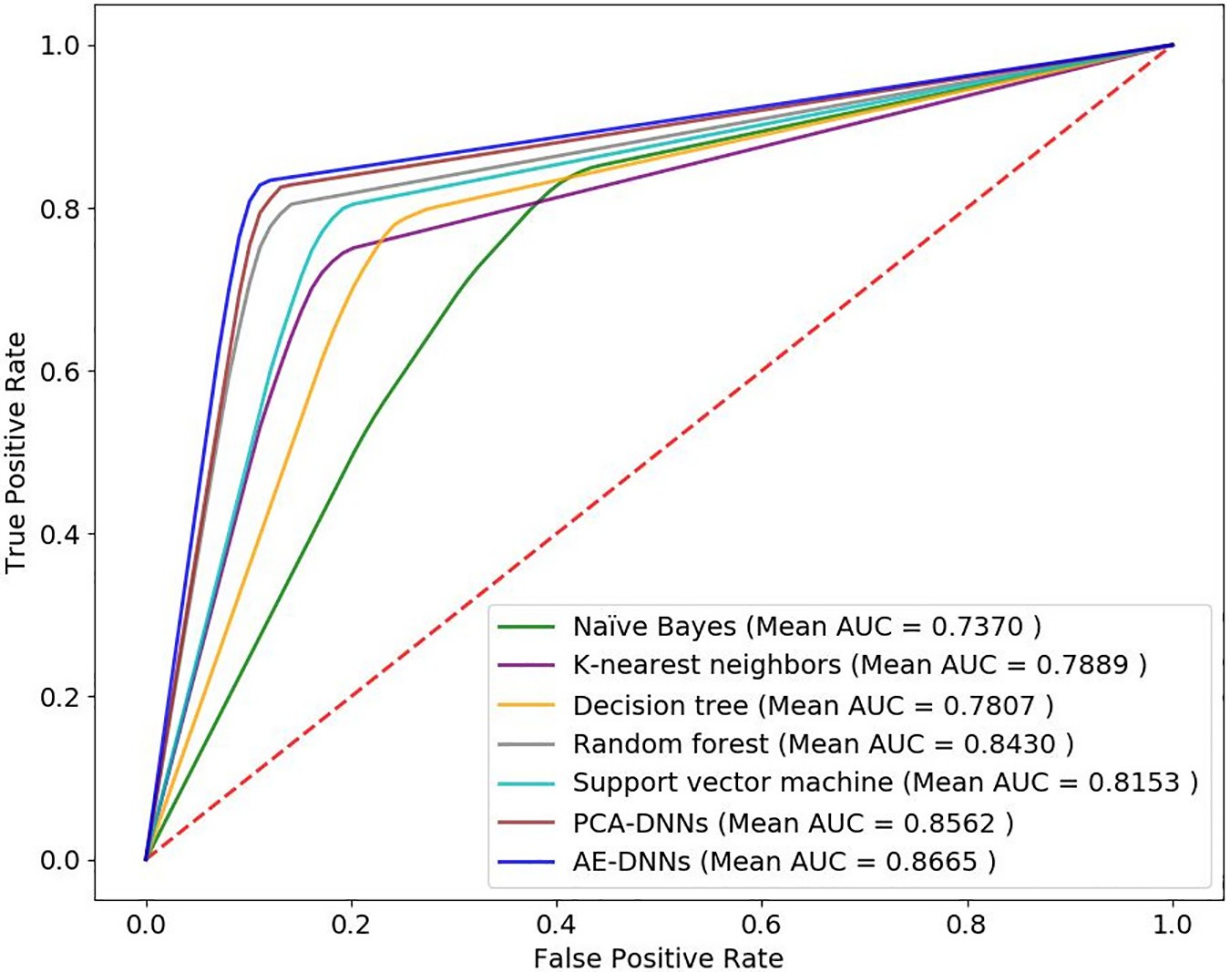
The average receiver operating characteristic curves of compared algorithms.

Fig 8 shows the average ROC curves of compared 7 methods. The AE-DNNs method shows higher AUC scores than other methods.

**Fig 9:**
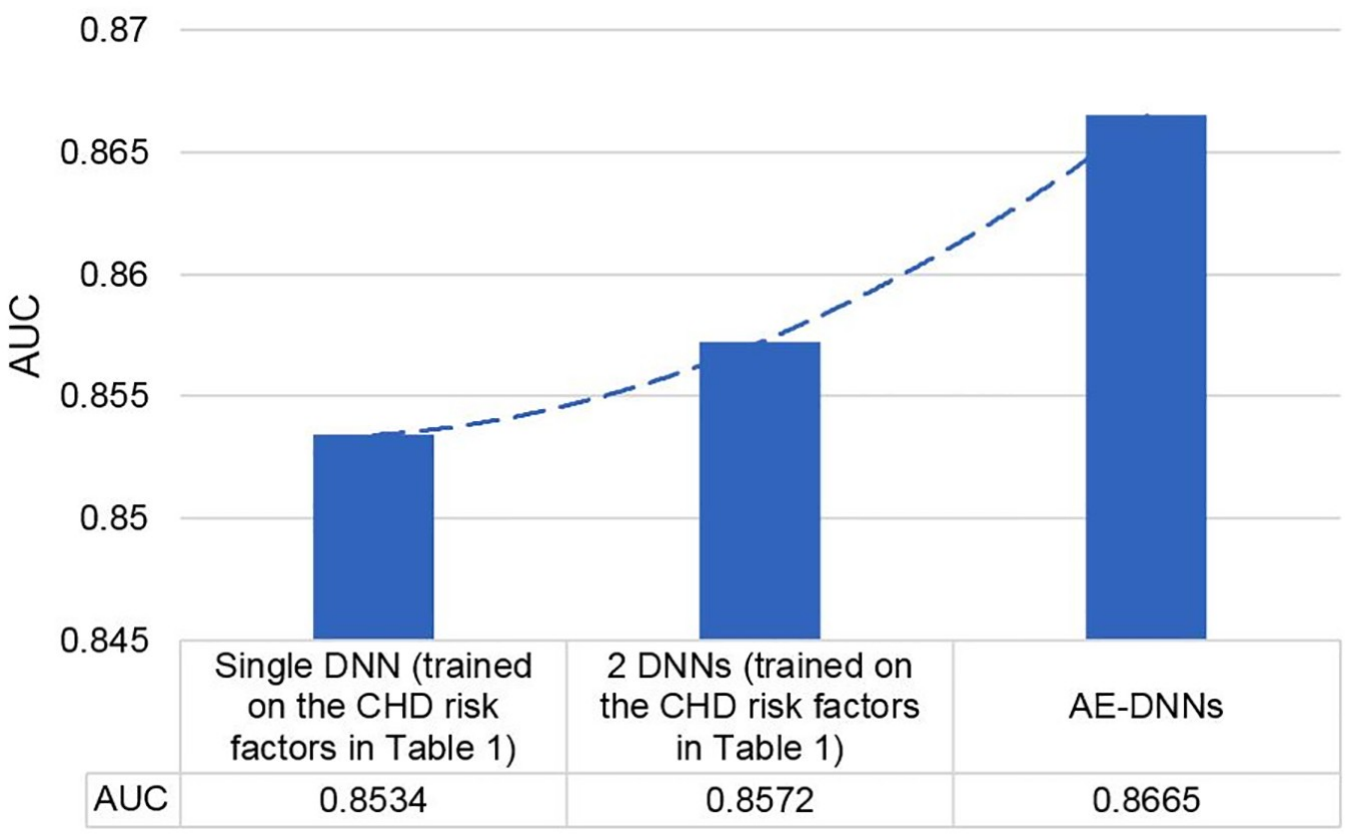
Improvement of area under the curve scores based on input features and the number of deep neural network classifiers.

Fig 9 shows how the AUC score of the proposed DNN was improved by managing two DNNs with the RE based feature. First, we trained a single DNN model using the selected features and received AUC of 0.8534. Second, we partitioned the training dataset into two groups based on their RE on the AE model that trained on the whole training dataset. Then, two DNN models learned from each of the groups, and its AUC score increased to 0.8572 from the single NN model. Finally, combining the RE based new feature with the selected CHD risk factors, AUC of the two DNNs based approach improved by 1.31% from the proposed single DNN model.

Experimental results show that we can use the AE-DNNs method for CHD risk prediction because it has given higher performance than other methods. In accordance with Tables 2 and 3, the RF algorithm had the highest accuracy, f-measure, and AUC scores from compared algorithms except for the proposed method, 84.42%, 84.99%, and 84.30% respectively. However, our proposed AE-DNNs has made these performances 86.33%, 86.91%, and 86.65%. Also, we changed AE modules by PCA modules, and the result of PCA-DNNs was higher than compared algorithms, but not well than AE-DNNs.

## Conclusions

In this paper, we have proposed a deep learning-based model to predict the risk of developing CHD and evaluated it in the Korean population. In the proposed method, two fully connected DNN classification models are combined with a deep AE models successfully. Generally, AE is used for dimensionality reduction. However, we did not use AE for dimensionality reduction purposes; it was employed as providing RE by projecting back reduced dimension into its original space. Two AE models named DAE-general and DAE-risky learned from the whole training dataset and the high-risk datasets, individually. First, RE based feature was extracted from the DAE-risky model, and it was used to feed the DNN model with other risk factors. Then, based on the RE of the DAE-general model, the whole training dataset was partitioned into two different subsets. Finally, two independent DNN classifiers were trained on each group; each group consists of the CHD risk factors and RE based newly created feature. In the prediction process, we compared the RE on the DAE-general model of each testing data to the previously determined threshold value and chose an appropriate classifier from these two DNN classifiers. By using two DNN classifiers with RE based feature, we improved the performance of single NN classifier on the whole dataset. Experimental results showed that the proposed AE-DNNs outperformed all the compared classifiers with accuracy, F-measure, and AUC score of 86.33%, 86.91%, and 86.65%, respectively.
